# College and Hospital Notes

**Published:** 1901-05

**Authors:** 


### COLLEGE AND HOSPITAL NOTES.

The German Hospital Dispensary staff has been reorganised. The following-named new members have been added: Dr. Frederick H. Millener, Dr. Julian A. Reister, Dr. Abram L. Weil, Dr. Frederick W. Koehler, Dr. Charles J. Mengis, Dr. Charles Filsinger, Dr. Edward A. Schweigert, Dr. Henry J. Siedler, Dr. Louis E. Beyer and Dr. Thomas Phillips.

---

The Buffalo General Hospital has issued its 42d annual report, which is for the year 1900. From it we learn that during that year there were treated in hospital 2,783 patients, of which 1,184 were medical, and 1,104 were general surgical cases. Besides these there were 15 ophthalmological, 49 otological and rhino-laryngological, and 431 gynecological patients treated or operated. The financial condition is strong, but it has a floating debt of $33,000, which has accumulated during the last five years. This is due to the fact that the city pays less than the cost of maintenance for the patients it sends to the hospital.

It has been calculated that the deficiency represents almost exactly $2 a week on the city patients that have been at the General Hospital during that time. So, if the city had paid $6 a week instead of $4 a week, the hospital would not now be facing a shortage of $33,000, although it would still have contributed to the poor in care and medical treatment nearly $45,000, since the expense per capita is over $9 a week, and one-third of that on all city patients would have been paid by the hospital.

This is an evil that the municipal government should make haste to correct.

---

The College of Medicine, Syracuse University, has a four years' course of study. Its facilities for instruction are unusually good. The main building, erected 1896, was carefully planned and constructed to meet in the most approved manner the requirements of a medical education of the present time. Competent judges pronounce this college unsurpassed in the state.

The New Orleans Polyclinic will, on account of various requests, continue its sessions to May 31, instead of May 11, 1901, as announced in the catalogue.

---

At the Buffalo General Hospital the changes in the resident staff are as follows: Dr. Edgar McGuire, senior surgeon, has been promoted to the position of house surgeon, to fill the vacancy that occurred upon the expiration of Dr. Alfred Zittel's term on May 1. Dr. Edwin R. Gould has been promoted to the position of house physician in place of Dr. William I. Thornton, whose position also expired on May 1. Dr. Charles Chance and Dr. David King will take the places made vacant by these promotions, while Dr. Tripp and Dr. Leonard will be junior surgeon and junior physician respectively.

---

The New York State Hospital for the Care of Crippled and Deformed Children, was opened in Tarrytown, December 1, 1900, and ten boys and girls are now there. The number of beds is limited to twenty-five. One of the difficulties connected with the state applicants lies in the fact that no funds are available to meet their traveling expenses. An effort to remove this obstacle was made in the last legislature.

Patients living at remote points of the state are referred to the following physicians, who are out-of-town members of the consulting staff: Drs. A. Vander Veer and S. B. Ward, of Albany, N. Y.; Dr. Louis A. Weigel, Rochester, N. Y.; Drs. Roswell Park and Charles G. Stockton, of Buffalo, N. Y., and Dr. Richard B. Coutant, of Tarrytown, N. Y.

